# Sex-Specific Muscle Size in Climbers: A Novel Cross-Sectional Study of an Ultrasonographic Analysis of Abdominal Wall Muscles

**DOI:** 10.3390/life15081275

**Published:** 2025-08-12

**Authors:** Harryson Emmanuel Caro-Betancur, Carlos Romero-Morales, Joel Zayas-Castaño, Diego Miñambres-Martín, Daniel López-López, Jorge Hugo Villafañe, Mónica García-Mateos

**Affiliations:** 1 Department of Physiotherapy, Faculty of Medicine, Health and Sports, European University of Madrid, Villaviciosa de Odón, 28670 Madrid, Spain; harrysoncarob05@gmail.com (H.E.C.-B.); joelzc01@gmail.com (J.Z.-C.); diego.minambres@universidadeuropea.es (D.M.-M.); mail@villafane.it (J.H.V.); monica.garcia@universidadeuropea.es (M.G.-M.); 2 Research, Health and Podiatry Group, Department of Health Sciences, Faculty of Nursing and Podiatry, Industrial Campus of Ferrol, Universidade da Coruña, 15403 Ferrol, Spain; daniellopez@udc.es

**Keywords:** ultrasonography, sport, public health

## Abstract

Aim: This study aimed to evaluate and compare the thickness of the external oblique (EO), internal oblique (IO), transversus abdominis (TrAb), rectus abdominis (RA), and the inter-recti distance (IRD) between male and female climbers, using B-mode ultrasonography to identify potential sex-based morphological differences. Results: Significant sex-based differences were observed in the thickness of several abdominal wall muscles—specifically the right IO, right RA, left EO, left IO, and left RA. No significant differences were found in the remaining muscles. Overall, male climbers exhibited greater muscle thickness than females, except for the left EO, which was thicker in the female group. Regression analysis showed that sex was a relevant factor associated with RA thickness and contributed, to a lesser extent, to the variability observed in both IO and EO muscles. Conclusion: This study highlights sex-related differences in abdominal muscle thickness among trained climbers. While male participants generally showed greater muscle thickness, the left EO was thicker in females. These morphological differences may reflect sex-specific anatomical adaptations, but further research is needed to determine their functional relevance. Current and future findings may contribute to the development of sex-specific training, rehabilitation, and injury prevention strategies in climbing.

## 1. Introduction

In recent decades, the popularity of various climbing disciplines—such as rock climbing and bouldering (a climbing modality performed at low height without rope protection)—has increased considerably [1]. During climbing, athletes perform complex movements at varying angles and heights, continuously controlling their own body weight through pulling, jumping, and using natural handholds [2]. Li L et al. reported that this sport modality requires a high physical preparation level, such as muscle power and endurance of the upper and lower limbs, feet, hands, and trunk muscles [2]. In this regard, Muehlbauer et al. demonstrated the benefits of an 8-week indoor training program on core and handgrip strength, as well as trunk mobility, in both male and female climbers [3]. The abdominal wall muscles play a crucial role in supporting and stabilizing the spine [4]. These muscles form a ring around the spine, composed of the external oblique (EO), internal oblique (IO), transversus abdominis (TrAb), and rectus abdominis (RA). They work in coordination with the diaphragm, lumbar multifidus, and pelvic floor muscles to transfer loads across the trunk and maintain balanced intra-abdominal pressure [4]. This synergy among muscles ensures better trunk control, efficient movement, proper balance, coordination, and improved motor control [5]. In addition to the previously mentioned benefits, structural data from healthy individuals indicate that the RA, IO, EO, and TrA account for approximately 35.0%, 28.4%, 22.8%, and 13.8% of the total abdominal muscle thickness, respectively. These proportions highlight the substantial contribution of each muscle to the overall structure of the abdominal wall [6].

The importance of the core muscles has been highlighted by Hodges et al., reporting functional deficits in subjects with lumbopelvic pain (LPP) [7]. In this context, muscle activity changes were found in core muscles in non-specific low back pain individuals [8], and a delay in the TrAb activation [9]. The abdominal wall muscles, commonly referred to as the “core”, should be considered a functional rather than solely anatomical unit [10]. Given the key role of these muscles in lumbopelvic stability and injury prevention, accurate tools are required to assess both their structure and function in clinical and athletic populations.

Several methods have been employed to assess the structure and function of the abdominal wall muscles, with electromyography (EMG) traditionally considered the gold standard technique. However, the use of ultrasound imaging (USI) has increased considerably, becoming a widely accepted alternative for real-time assessment of the morphology and texture of core muscles. A systematic review conducted by Taghipour et al. supported the use of USI, reporting good levels of both intra- and inter-rater reliability for evaluating trunk stabilizer muscles [11]. The recent literature has demonstrated the usefulness of USI for examining abdominal wall muscles in both athletic and non-athletic populations, using static and dynamic B-mode imaging [12,13,14,15]. Moreover, several authors have emphasized the importance of conducting quantitative and standardized assessments of trunk muscles using USI. Therefore, ultrasonography is currently considered a fast, portable, and relatively cost-effective tool that allows for accurate real-time evaluation [16].

However, despite the growing popularity of climbing and the recognized importance of core muscles in athletic performance and injury prevention, there is limited research evaluating the abdominal wall muscles in climbers, particularly regarding potential anatomical differences (such as thickness) and functional variations between sexes. Understanding these differences is essential, as they may significantly influence biomechanical efficiency, performance, and injury risk in this high-intensity sport. Furthermore, this knowledge is crucial for designing sex-specific training and rehabilitation protocols tailored to the physiological needs of competitive climbers. This gap in the literature highlights the need for objective, real-time assessments using reliable tools such as USI.

Therefore, the aim of the present study was to evaluate and compare, using B-mode ultrasound, the thickness, and inter-recti distance (IRD) of the abdominal wall muscles (EO, IO, TrAb, and RA) between male and female climbers.

## 2. Methods

### 2.1. Design

A cross-sectional observational study was developed according to the Strengthening the Reporting of Observational Studies in Epidemiology (STROBE) guidelines [17]. Data were collected between March 2024 and May 2024.

### 2.2. Sample Size Calculation

The sample size calculation was performed by the differences between 2 independent groups with G*Power 3.1 software and based on IO thickness of a pilot study (*n* = 16) with 2 groups (mean ± SD): 8 male (11.8 ± 2.85 cm) and 8 female (9.55 ± 2.71 cm) climbers. For the sample size calculation, 1-tailed hypothesis, an effect size of 0.80, alpha error probability of 0.05, a power of 0.80, and an allocation ratio (N1/N2) of 1 were employed. Thus, a total sample size of 40 individuals, 21 for each group, was considered.

### 2.3. Ethical Considerations

The present study was approved by Universidad Europea Research Ethics Committee (CIPI 2024-559), and participants signed the informed consent form before the beginning. The study also adhered to the ethical standards of the Declaration of Helsinki for human experimentation [18].

### 2.4. Participants

A total sample of 40 healthy climbers was recruited through convenience sampling at two climbing facilities in the Community of Madrid, Spain. Climbers were invited to participate, and those who agreed and met the selection criteria were included in the study. The sample was divided into two groups: male group (*n* = 20) and female group (*n* = 20). Climbers followed a training schedule for 2 h each day, 5 days per week. Inclusion criteria were age between 18 and 40 years, being in good health during data collection, and having had no injuries in the previous three months. Exclusion criteria were BMI greater than 30 kg/m^2^ [14], any musculoskeletal condition, neurological disorders, skin diseases of the abdominal region, systemic disturbances [6], previous surgery or abdominal hernia [19], upper or lower limb pathology (e.g., fractures, osteoarthritis), women who had previously given birth were excluded (reason for exclusion of a participant for the female group), and allergic reactions to the ultrasound gel [20].

### 2.5. Ultrasonography Measurements

**Figure 1:**
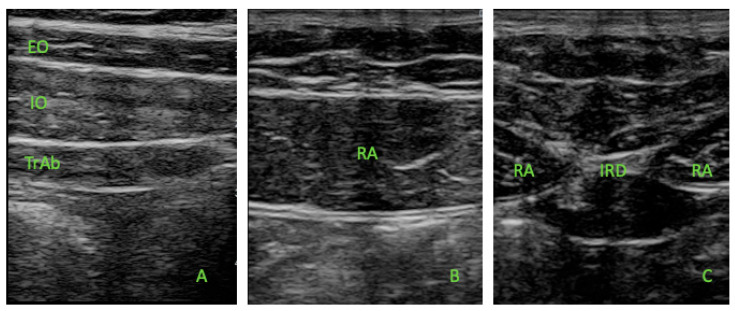
Ultrasound thickness assessments of the EO, IO, TrAb (**A**), RA (**B**), and IRD (**C**).

All measures were performed by the same therapist (J.Z.) with experience in USI. An ultrasound system (SonoScape E2, SonoScape ES) was used in B-Mode for ultrasound imaging assessment. All the participants were placed in a supine position for the data collection. Muscle thickness was defined as the distance between the edges of each muscle border. Moreover, considering prior studies about USI measurements in the abdominal wall muscles, we exclude the perimuscular and fascial connective tissues [21]. The image analysis was not performed in a blinded manner, and inter- or intra-rater reliability was not assessed. However, ultrasound images were recorded following the Teyhen et al. recommendations [16], ultrasonography for the EO, IO, and TrAb muscles was performed by placing the probe between the subcostal line and the iliac crest and the mid-axillary line (Figure 1A). The RA muscle assessment was carried out with the transducer aligned with the umbilicus, and just under the umbilicus for the IRD measurement. (Figure 1B,C). The mean of two repeated measures was recorded for each measurement, with the probe located at the same place with slight pressure on the skin (pressure generated by the transducer weight) at the end of the expiration. All the images were assessed on both sides in all the participants.

### 2.6. Statistical Methods

Analyses were conducted using RStudio (v.1.4, RStudio, PBC, Boston, MA, USA) and Jamovi (v.1.6, The jamovi project). Data were tested for normality employing the Shapiro–Wilk test (*p* > 0.05). Descriptive data were described using mean and standard deviation (SD) for parametric data and median and interquartile range (IQR) for non-parametric data. For the comparative analysis of parametric data for both groups, Student’s *t*-test for the independent samples was used, whereas for non-parametric data analysis, the Mann–Whitney *U* test was used. Moreover, Levene’s test was used to check the homogeneity of variances. The effect size between groups was estimated using Cohen’s *d*, interpreting values of 0.2 as small, 0.5 as medium, and 0.8 as large effects. Additionally, a multivariate linear regression analysis (stepwise method; Pin = 0.05, Pout = 0.10) was conducted to identify predictors of abdominal muscle thickness. The dependent variables were the muscles with significant group differences (RA, IO, and EO). Independent variables included sex, age, weight, height, and BMI. This approach allowed us to assess the individual contribution of each variable to muscle thickness while controlling for potential confounders.

## 3. Results

### 3.1. Descriptive Analysis

Significant sex differences were found in sociodemographic variables, including weight (*p* < 0.001), height (*p* < 0.001), and body mass index (BMI; *p* = 0.016) (Table 1).

### 3.2. Group Comparisons

Regarding abdominal wall muscle thickness (Table 2), males demonstrated significantly greater thickness than females in the right internal oblique (IO; *p* = 0.013), right rectus abdominis (RA; *p* = 0.001), left IO (*p* = 0.003), and left RA (*p* = 0.001). Interestingly, the left external oblique was significantly thicker in females (*p* = 0.031). No other significant differences were found in the remaining muscles.

### 3.3. Regression Analysis

Linear regression analyses (Table 3) revealed that sex was a significant predictor of muscle thickness for the left RA (*R*^2^ = 0.716) and right RA (*R*^2^ = 0.629; *p* < 0.05). For the right IO, left IO, and left EO muscles, sociodemographic variables and sex explained 24%, 32%, and 21% of the variance in thickness, respectively.

## 4. Discussion

To our knowledge, this study is the first to explore differences in abdominal wall muscles between sexes among trained climbers. Our findings revealed differences in muscle thickness, with greater thickness observed in the left and right IO and RA muscles in male participants compared to females. In contrast, the left EO muscle was thicker in the female group.

Interestingly, this greater thickness in the left EO among female climbers contrasts with the general pattern observed in the other abdominal muscles. This atypical result may be related to side dominance or asymmetrical loading patterns during climbing, which could differ by sex. It may also reflect compensatory activation or technique-specific adaptations in female climbers. Although this study focused on muscle thickness, future research should explore how these anatomical characteristics influence climbing-specific tasks, such as sustained isometric core contractions, trunk rotation, and postural control during complex movement sequences. Considering the distinct physical demands of different climbing disciplines [2], these factors may help explain sex-specific adaptations in trained climbers, although this remains hypothetical and was not directly assessed in the present study. Moreover, techniques such as drop-knees, heel hooks, and high steps often involve asymmetrical loading and side-dominant strategies, which could contribute to the morphological differences observed in this study [22].

Ultrasound imaging in this study provided a reliable, non-invasive method for assessing abdominal wall muscle thickness. Previous research supports its validity for both static and dynamic assessments, showing high intra- and inter-rater reliability in trunk musculature evaluation [11]. Its real-time imaging capability and portability make ultrasound particularly suitable for studying athletic populations—such as climbers—in both clinical and field settings, thus contributing to the generalizability of our findings [12,13,14,15,16].

In line with our results, Rho et al. reported that asymptomatic men had greater IO thickness at rest compared to women, although women demonstrated greater percent change in TrAb thickness during the draw-in maneuver [23]. Another important aspect of abdominal wall muscles between the sexes involves anatomical differences. For example, the IO is connected to the fascia of the cremaster muscle in men, but this relationship does not exist in women. For this reason, training and development of this musculature can offer slightly different benefits between men and women [24]. Da Cuña-Carrera et al. argued that TrAb and EO increased their thickness during hypopressive exercise, with higher effects in men, and IO showed higher effects in women. Thus, it can be established that the unequal core muscles anatomy could explain the different values between sexes [24].

Several authors support that sex differences occur during athletic maneuvers such as jumping [25], landing [26], and performing functional tasks [27,28,29].

Greene et al. suggested that core stability differences between sexes can influence energy transfer efficiency. They also noted that males generally have higher testosterone levels, contributing to greater muscle mass and power output [30]. Additionally, differences in muscle stiffness and elasticity between sexes may affect how these muscles function during climbing activities [31]. However, these aspects were not assessed in the present study and should be interpreted as hypothetical within our context.

Moreover, Carroll et al. noted that the exceptional ability of female climbers serves as further evidence of sex-blind musculoskeletal adaptations that enhance the capacity for essential movements [32]. This perspective is interesting, although our morphological findings cannot confirm functional or performance-related advantages.

Regarding lateral abdominal muscles, Niewiadomy et al. found no significant sex differences in TrAb thickness or trunk mobility [33]. In contrast, Lim et al. reported significantly greater EO and IO muscle thickness in males [34].

The activation of the TrAb muscle in female athletes—with no significant differences found in our study but results comparable to those of the male group—could reflect consistent core demands placed on this muscle during training and climbing. Therefore, adequate motor control and maintenance of TrAb muscle thickness in both sexes may be considered a protective factor against urinary incontinence in female athletes [12].

As part of the statistical approach, we included regression analyses to examine the potential influence of sex on abdominal muscle thickness. While sex was identified as a relevant factor in explaining the variability of RA thickness, the models for other abdominal muscles demonstrated low explanatory power (*R*^2^ < 0.3), limiting the strength of these associations. These interpretations should be approached with caution due to several methodological limitations. The sample size was calculated based on a small pilot study with a relatively large, assumed effect size, which may have led to an overestimation of statistical power. Furthermore, no intra- or inter-rater reliability assessments were conducted, which could affect the consistency of the ultrasound measurements. Taken together, these factors limit the robustness of the statistical conclusions and underscore the exploratory nature of the findings, highlighting the importance of future research with larger samples and improved methodological rigor.

These findings suggest that sex-specific anatomical characteristics may play a role in how core muscles are structured in trained climbers. While such morphological differences could have implications for training, rehabilitation, and injury prevention, our data do not allow us to draw conclusions about functional performance or clinical outcomes. Further research—particularly studies incorporating functional, biomechanical, and longitudinal data—is needed to determine whether and how these anatomical features influence climbing-specific movement, performance, or injury risk. Until such evidence is available, these findings should be interpreted as exploratory and hypothesis-generating.

### 4.1. Clinical Applications

Since the findings of this study are based exclusively on morphological assessments, any potential clinical applications should be interpreted as hypothetical at this stage. Nevertheless, evaluating the abdominal wall muscles could, in the future, contribute to the development of rehabilitation and training programs aimed at improving core stability, mobility, and efficient load transfer from the lower to the upper limbs. In this context, ultrasonography may serve as a valuable non-invasive tool for monitoring muscle changes over time and supporting future injury prevention strategies. However, both functional and longitudinal validation are needed before drawing definitive clinical implications. This perspective is supported by previous studies that have demonstrated the clinical utility of ultrasound in athletic populations [35], as well as its applicability in assessing abdominal wall musculature [36].

### 4.2. Limitations

This study has several limitations that should be considered. First, the sample size was relatively small. Although a power analysis was performed, it was based on a pilot study that used a large effect size (d = 0.80), which may have led to an overestimation of statistical power. Second, measurements were taken at a single static time point; assessing abdominal wall muscles during dynamic movements would offer a more comprehensive understanding of their function. Third, the ultrasound image analysis was not blinded, as the evaluator was present during real-time image acquisition and was necessarily aware of the participant’s sex. In addition, no formal assessment of intra- or inter-rater reliability was conducted. Although all measurements were performed by the same experienced evaluator following standardized protocols described in previous studies, this approach does not replace formal reliability testing. Finally, the cross-sectional design limits the ability to draw causal inferences between core muscle characteristics and climbing performance or injury risk.

### 4.3. Future Lines

Future studies should consider weight-bearing ultrasonography to observe abdominal muscle activity under real biomechanical conditions. Longitudinal studies are needed to establish causal relationships between muscle architecture and an athlete’s climbing performance or injury over time. Therefore, it could be beneficial to compare muscle changes among climbers of different expertise levels in different types of climbing. For instance, bouldering involves short, explosive routes without ropes, whereas lead climbing requires longer routes with higher endurance demands and rope use. These differences may create distinct muscular demands and activation patterns that deserve separate study.

## 5. Conclusions

This study presents new evidence of sex-related differences in abdominal muscle thickness among climbers. Ultrasonography results reported that male participants generally exhibited greater muscle thickness across most measured abdominal muscles, except for the left EO, which was thicker in female participants. These results are consistent with established anatomical and physiological differences between sexes and highlight the potential influence of biological factors such as hormone levels and the mechanical properties of muscle tissue.

The observed differences in muscle architecture suggest potential sex-based anatomical adaptations. While these differences may influence how core muscles are recruited during climbing, their effect on performance and injury risk remains to be investigated with functional and biomechanical data. For example, greater muscle thickness in males might confer mechanical advantages for power and load transfer, whereas females might benefit from enhanced flexibility, aiding technical maneuvers. However, these interpretations should be considered hypothetical, as they were not directly assessed in the present study and warrant further investigation in future research.

Considering these anatomical differences, future training, rehabilitation, and injury prevention programs may benefit from considering sex-specific characteristics. However, functional outcomes should be explored in future studies before establishing clinical recommendations.

## Figures and Tables

**Table 1:** Sociodemographic data.

Measurement	Male (*n* = 20) Mean ± SD or Median (IQR)	Female (*n* = 20) Mean ± SD or Median (IQR)	*p*-Value
Age, y	36.5 ± 10.8 *	35.0 ± 9.60 *	0.656 **
Weight, kg	72.0 (9.0) ^†^	57.0 (10.0) ^†^	0.001 ^‡^
Height, cm	1.77 ± 0.0 *	1.65 (0.0) ^†^	0.001 ^‡^
BMI, kg/cm^2^	23 (3) ^†^	21 (2) ^†^	0.007 ^‡^
Discipline, B/R/M	4/3/14	5/5/11	n/a
Experience, y	4	4	n/a
Shoe size	43 (0.5) ^†^	39 ± 1.22 *	n/a

Abbreviations: B, boulder; R, rope; M, mixed. * Mean (SD) was applied. ** Student’s *t*-test for independent samples was performed. ^†^ Median (IQR) was used. ^‡^ Mann–Whitney U test was utilized.

**Table 2:** Ultrasound imaging measurements of the abdominal wall muscles.

Measurement	Male (*n* = 20) Mean ± SD (95%CI) or Median (IQR)	Female (*n* = 20) Mean ± SD (95%CI) or Median (IQR)	*p*-Value (Effect Size)
TrAb_R	4.32 (3.2–8.0) ^†^	4.42 ± 1.14 (2.4–7.6) *	0.636 (0.18) ^‡^
IO_R	11.6 ± 2.56 (6.6–19.2) *	9.40 (3.8–19.6) ^†^	0.013 (0.62) ^‡^
EO_R	6.87 ± 1.52 (4.7–10) *	6.33 ± 1.48 (4.0–9.6) *	0.274 (0.35) **
RA_R	14.7 ± 2.41 (11.1–20.8) *	9.69 ± 1.81 (6.9–13.5) *	0.001 (2.32) **
TrAb_L	4.72 (2.7–8.3) ^†^	4.23 ± 1.32 (2.3–6.8) *	0.379 (0.27) ^‡^
IO_L	11.5 ± 2.54 (7.6–18.4) *	9.64 (5.66–11.6) ^†^	0.003 (1.08) ^‡^
EO_L	6.64 (4.29–13.4) ^†^	7.18 ± 2.14 (2.76–9.50) *	0.031 (0.66) ^‡^
RA_L	13.5 ± 2.06 (10.8–17.7) *	9.44 ± 1.94 (6.29–13.2) *	0.001 (2.0) **
IRD	5.31 ± 1.87 (1.8–9.7) *	4.70 (1.9–10.2) ^†^	0.365 (0.26) ^‡^

Abbreviations: EO, external oblique; IO, internal oblique; IRD, inter-recti distance; RA, rectus abdominis; TrAb, transversus abdominis. * Mean (SD) was applied. ** Student’s *t*-test for independent samples was performed. † Median (IQR) was used. ‡ Mann–Whitney U test was utilized.

**Table 3:** Multivariate predictive analysis for RA, IO, and left EO significative values.

Parameter	Model	*p* Value	Model *R*^2^
RA_R (cm)	10.750		0.716
	−0.11 * Age	0.001	
	−0.09 * Weight	0.177	
	−0.02 * Height	0.412	
	−0.19 * BMI	0.359	
	−4.22 * Sex (Female − Male)	0.001	
RA_L (cm)	18.247		0.629
	−0.07 * Age	0.034	
	0.008 * Weight	0.196	
	0.01 * Height	0.687	
	0.06 * BMI	0.750	
	−2.76 * Sex (Female − Male)	0.003	
IO_R (cm)	15.730		0.245
	0.05 * Age	0.058	
	0.09 * Weight	0.001	
	−0.10 * Height	0.296	
	−0.54 * BMI	0.283	
	−1.25 * Sex (Female − Male)	0.351	
IO_L (cm)	10.277		0.322
	0.05 * Age	0.203	
	0.07 * Weight	0.339	
	−0.05 * Height	0.139	
	−0.26 * BMI	0.309	
	−1.63 * Sex (Female − Male)	0.133	
EO_L (cm)	4.274		0.215
	−0.04 * Age	0.195	
	0.05 * Weight	0.423	
	−0.01 * Height	0.744	
	0.02 * BMI	0.909	
	−0.34 * Sex (Female − Male)	0.698	

Abbreviations: EO, external oblique; IO, internal oblique; RA, rectus abdominis.

## Data Availability

The original contributions presented in the study are included in the article, further inquiries can be directed to the corresponding author.
